# Editorial: Case reports in molecular and cellular oncology: 2022

**DOI:** 10.3389/fonc.2023.1209048

**Published:** 2023-05-16

**Authors:** Shuhua Zheng, Tarita O. Thomas

**Affiliations:** Department of Radiation Oncology, Robert H. Lurie Comprehensive Cancer Center of Northwestern University, Chicago, IL, United States

**Keywords:** targeted therapy, molecular marker, radiation, lung cancer, personalized medicine

Large scale clinical trials clearly demonstrate overall survival benefits of targeted therapies in many human malignancies. This Research Topic in Molecular and Cellular Oncology demonstrated the unique challenges related with targeted therapy when disease evolves under selection pressure from targeted therapies. Li et al. reports an interesting evolutionary process of EGFR alteration patterns when a stage IV lung adenocarcinoma patient is under selective pressure while on a TKI. The resistance mechanism involves enrichment of differentially mutated or abnormally amplified EGFR. The patient has relatively extended stable disease periods when a TKI is tailored based on specific EGFR alterations from biopsies at different stages of disease progression. Accumulation of novel mutations during the disease course may exhaust options of targeted therapies. Case reports in this Research Topic show off-label use of TKIs for novel EGFR mutations may have unexpected therapeutic benefits as demonstrated by other cohort studies (Li et al., 1).

Safety and efficacy of combined treatment of targeted therapy and radiotherapy is not well reported in clinical trials. This Research Topic showed several cases with good outcomes when concurrent or sequential treatment of radiation and targeted therapeutic agents (Li et al.; Sun et al.). For example, a radiographic complete response was achieved with concurrent chemo-/radio-therapy and crizotinib for a rare locally-advanced gingival sarcomatoid squamous cell carcinoma patient with MET exon 14 skipping mutation (Sun et al.). Interestingly, Li et al. reported management of a intracranial lesion for an EGFR-mutated NSCLC patient with radiation followed by adaptive targeted therapies. They reported the patient had unexpectedly good clinical response with intracranial disease control of 23 months.

This series of articles in Molecular and Cellular Oncology highlights the gaps in understanding how targeting a single mutation alters the cellular microenvironment and implications of this as patients are living longer on these therapies. Further studies are needed to better understand genetic alterations in tumor response to novel targeted therapies and their interactions with chemo-/radio-therapies.

## Author contributions

SZ drafted the editorial; TT edited and revised the editorial. All authors contributed to the article and approved the submitted version.
